# Global inequality in sub-fertility treatment needs safer, cost effective, evidence-based and economically viable choices for patients and stakeholders

**DOI:** 10.5935/1518-0557.20210111

**Published:** 2022

**Authors:** Gulam Bahadur, Roy Homburg, Asif Muneer, Paul Racich, Kanna Jayaprakasan, Santanu Acharya, Eric Jauniaux

**Affiliations:** 1 Reproductive Medicine Unit, North Middlesex University Hospital, Old Admin Block, London N18 1QX, UK; 2 Homerton Fertility Centre, Homerton University Hospital, London E9 6SR, UK; 3 NIHR Biomedical Research Centre University College London Hospital, London NW1 2BU, UK; 4 Linacre College, Oxford University, St. Cross Road, Oxford, OX13JA, Oxfordshire, UK; 5 Derby fertility unit, Royal Derby Hospital, Derby, UK; 6 Ayrshire Fertility Unit, University Hospital Crosshouse, Kilmarnock, Scotland, UK; 7 EGA Institute for Womens Health, Faculty of Population Health Science, University College London, London, WC1E 6HX, UK

**Keywords:** IVF, IUI, ICSI, cost-effectiveness, fertility industry, health-economics

## Abstract

The global increase in subfertility diagnosis and treatments and the rise of private equity investors concentrating on high profits based on *in vitro* fertilisation (IVF) treatments raise profound societal and economic questions for stakeholders and patients. The question remains as to whose benefits will ultimately be greater when promoting high margins treatment options resulting from cross-border mergers and acquisitions of IVF clinics.This paper covers wide-ranging issues from the erroneously constructed UK National Institute for Health and Care Excellence’s (NICE) guidelines on treatment choices, the cost-effectiveness of treatments, the promotion of IVF, and add-ons where evidence remains minimal, the commercial size of the fertility industry. Investment in improving intrauterine insemination (IUI) success rates has understandably been avoided for its short-term impact on the IVF industry. However, IUI efficiency would cut across many of the global subfertility treatment economic and access problems while allowing stakeholder, feepaying, and patients financial savings will likely allow for more funded IVF cycles in acutely deserving cases. The recommendations will help expand choices for globally economically challenged patients’ and services while enhancing an ethical and moral dimension towards fertility treatment choices for patients and stakeholders.

## BACKGROUND

Global sub-fertility treatments centre around expensive IVF treatments with successes of only 30% meaning 70% of women fail to have a baby, despite cumulative success claims (Ekechi, 2021; Rienzi et al., 2021; Bahadur et al., 2020; Spencer et al., 2016). The UK NICE’s non-evidenced-based guidelines relating to IUI and IVF remain unchanged despite an expected 2017 review Bahadur et al. (2017). The IVF industry is attracting unprecedented private investment witnessed by the current mergers and acquisitions of IVF clinics (Willems, 2021). This raises concerns for patients and stakeholders’ interest in the UK fertility industry currently worth £320 million per annum, with minimum 10% margins (HFEA, 2019), against a projected $40-billion global market including cryopreservation of gametes and embryos (The Economist, 2019). Mergers are primarily associated with asset-stripping, staff optimisation, financial gain for shareholders, and neutralising competition.

## ECONOMIC AND SAFETY EVIDENCE

The cost-effectiveness of an IUI live birth (LB) is £42,000 cheaper than a LB through IVF, and these savings can be extended to £76,000/LB in well-managed clinics (Bahadur *et al*., 2020). NICE has yet to inform stakeholders of the cost-effectiveness of IUI. Contrary to the public exercise of blaming IUI for unchecked multiple gestation pregnancies (MGP), the risk remains with IVF while accruing an NHS MGP cost burden of around £120 million per annum, leaving the profits to the IVF industry. No other industry can pass on the risk burden to the state while keeping the profits only. ICSI instead of IVF is disproportionately used without evidence in order to optimise profits and has become the primary therapeutic approach for couples with `unexplained infertility (Dang *et al*., 2021). Technology creep has disproportionately increased `add-ons,’ while the cryopreservation industry with 3.1-9.3% usage of frozen material sets the cost of each extra LB between $600,000-1,000,000 (Ben-Rafael, 2018).

## THE FUTURE

The unregulated growth of the IVF and cryopreservation industries, spurred on by the private sector now requires re-focusing NHS budgets towards lesser intrusive and safer IUI procedures where the common objective is achieving a healthy LB, while reducing maternal and neonatal health risks and cost burdens. CCGs and the NHS would do well to prioritise IUI over IVF and ICSI to ensure all subfertile patients can receive appropriate NHS treatment. 

## CONCLUSION

With the current evidence on the cost-effectiveness of IUI there is no reason why this should not be practiced in most cases where severe male factor or bi-lateral tubal blockage is not involved. This will help cost savings to allow for deserving cases to be treated with more IVF and then ICSI cycles. The proposals allow for a sound ethical, and moral framework in an industry disproportionally focussed on profits, which allows a degree of protection to patients and fee-paying stakeholders, while addressing the global access problem in economically challenged countries. The fertility industry has a duty of care to improve on IUI outcomes and encourage a greater provision of alternative less intrusive and safer options.
